# High-speed femtosecond laser plasmonic lithography and reduction of graphene oxide for anisotropic photoresponse

**DOI:** 10.1038/s41377-020-0311-2

**Published:** 2020-04-26

**Authors:** Tingting Zou, Bo Zhao, Wei Xin, Ye Wang, Bin Wang, Xin Zheng, Hongbo Xie, Zhiyu Zhang, Jianjun Yang, Chunlei Guo

**Affiliations:** 1 State Key Laboratory of Applied Optics, Changchun Institute of Optics, Fine Mechanics and Physics, Chinese Academy of Sciences, 130033 Changchun, China; 2 Center of Materials Science and Optoelectronics Engineering, University of Chinese Academy of Sciences, 100049 Beijing, China; 3 Department of Electronic Information and Physics, Changzhi University, 046011 Changzhi, China; 4 Key Laboratory of Optical System Advanced Manufacturing Technology, Changchun Institute of Optics, Fine Mechanics and Physics (CIOMP), Chinese Academy of Sciences (CAS), 130033 Changchun, China; 5 The Institute of Optics, University of Rochester, Rochester, NY 14627 USA

**Keywords:** Ultrafast lasers, Nanophotonics and plasmonics

## Abstract

Micro/nanoprocessing of graphene surfaces has attracted significant interest for both science and applications due to its effective modulation of material properties, which, however, is usually restricted by the disadvantages of the current fabrication methods. Here, by exploiting cylindrical focusing of a femtosecond laser on graphene oxide (GO) films, we successfully produce uniform subwavelength grating structures at high speed along with a simultaneous in situ photoreduction process. Strikingly, the well-defined structures feature orientations parallel to the laser polarization and significant robustness against distinct perturbations. The proposed model and simulations reveal that the structure formation is based on the transverse electric (TE) surface plasmons triggered by the gradient reduction of the GO film from its surface to the interior, which eventually results in interference intensity fringes and spatially periodic interactions. Further experiments prove that such a regular structured surface can cause enhanced optical absorption (>20%) and an anisotropic photoresponse (~0.46 ratio) for the reduced GO film. Our work not only provides new insights into understanding the laser-GO interaction but also lays a solid foundation for practical usage of femtosecond laser plasmonic lithography, with the prospect of expansion to other two-dimensional materials for novel device applications.

## Optoelectronics: Ultrafast lithography technique lays foundations for novel 2D materials

A simple, versatile, and high-speed technique for producing uniform, large-area micro- and nano-sized gratings on graphene oxide (GO) films could pave the way for new materials for use in a range of optoelectronic devices. Although there are several techniques for fabricating structures on GO films, including directed-assembly and nanoimprint and lithography combined with electron-beam etching, they are either time-consuming, costly or lack versatility. Led by Jianjun Yang from the Changchun Institute of Optics, Fine Mechanics and Physics, a team of Chinese researchers has used ultrafast femtosecond laser plasmonic lithography (FPL) to create subwavelength grates on GO films 140 nanometres thick. The work has, for the first, demonstrated the practical use of FPL for producing structures on two-dimensional films of GO for use in novel devices for applications in optoelectronics, such as energy harvesting, and chemical and biological sensing.

## Introduction

Graphene analogs, such as graphene oxide (GO) and its reduced forms (rGO), are fascinating carbon materials due to the complementary properties endowed by the *sp*^3^-*sp*^2^ interconversion, revealing the substitutability and potential for industrialization of integrated graphene devices^1,2^. Reasonable micro/nanostructural design of GO and rGO to control the energy band gap and surface chemical activity is important for developing strategic applications^3,4^. In particular, uniform periodic structures over large areas exhibit attractive collective oscillations of electrons in plasmonic systems, which enable the patterned materials to have great vitality in numerous research areas, such as optical field regulation, optoelectronics, energy harvesting and chemo/biosensing^5–8^.

Effective implementation of many techniques, including directed assembly, nanoimprinting, and lithography combined with electron-beam etching, thermochemical, and scanning probe technologies, for fabricating structures with high spatial resolution has been demonstrated, but these techniques are time consuming, costly, or lack versatility^9–13^. In contrast, femtosecond laser-based processing, associated with the benefits of being facile, maskless, and efficient, is a qualified candidate for industrial development^14^, and recently, it has been applied in the micro/nanomanufacturing of graphene analogs, such as typical patterning of GO films^15^. Due to the nonthermal and thermal effects during this processing, simultaneous in situ photoreduction can be realized just by removing the oxygen-containing functional groups of GO, which leads to controllability of GO’s morphology and reduction degree by changing the duration, intensity, or wavelength of the laser irradiation^15–17^. At present, the most commonly adopted technologies for photoreduction of GO materials by femtosecond lasers are based on direct writing and beam interference^18,19^. The difficulty faced in the former case is the slow processing speed, while the latter’s greatest problem is the poor splicing quality of the micro/nanostructures formed at the joint of the moving light spots, especially when a large area must be processed. Meanwhile, confined by the optical diffraction limit, the maximum processing accuracy of both common methods can only be comparable to the incident wavelength.

Here, by utilizing a new strategy of femtosecond laser processing, so-called femtosecond laser plasmonic lithography (FPL), we present high-speed, large-area micro/nanomanufacturing, and photoreduction of GO films (~140 nm) on a silicon substrate (300 nm SiO_2_/Si). Periodic grating structures with high regularity (~680 nm period) can be induced on the surface of the reduced GO film even at a centimeter scale. Although the first observation of laser-induced periodic surface structures (LIPSSs) can be traced back to 50 years ago^20,21^, whose universal property has been confirmed for various materials, including metals, semiconductors, and insulators, the low structural quality is obviously one of the most important factors restricting their further application. Flexible and controllable energy deposition to achieve practical structural fabrication in the subwavelength range through the FPL strategy has only been recognized in recent years^22–26^. The key factor in this process is the lossy and reversible nature of the plasma waves, which allows us to obtain feedback at local positions and in turn control the energy distribution to achieve the formation of regular structures^22,27^. In our experiments, we demonstrate that the laser-induced gradient reduction of GO film from its surface to the interior plays a key role, which produces an inhomogeneous slab with the maximum dielectric permittivity (DP) at the surface and a smaller DP at deeper thicknesses that allows excitation of TE-mode surface plasmons (TE-SPs)^28–30^. The further interference between the incident light and the excited TE-SPs modulates the laser energy distribution, leading to a periodic LIPSS with orientation parallel to the laser polarization direction^23,27^. Notably, the idea of TE-SP excitation is newly proposed in the application of the FPL strategy, which obviously helps us understand the similar processing of related materials.

Different from previous reports about the laser processing of graphene materials, our manufacturing process has diverse physical mechanisms during the laser–rGO interaction and exhibits unique characteristics, such as high efficiency and strong robustness against a range of perturbations. The simultaneous occurrence of the large-area grating structure formation and photoreduction processes greatly simplifies the work procedures. For example, compared to laser direct writing adopting the same incident laser parameters, the FPL strategy takes only ~1/14,000 of the time to process a centimeter-sized sample (1 × 1.2 cm^2^). Meanwhile, due to the possible nonlinear optical property^22^, the FPL strategy induces an obvious “self-repairing” phenomenon, which can effectively guarantee the processing quality. We can prepare rGO-LIPSS films on different substrates and nondestructively transfer them onto other substrates. In addition, as another new phenomenon, the processed grating structure strictly parallel to the TE polarization state of the incident light should also be noted. This implies that complex topography preparation is feasible by changing only the polarization^23^.

All the abovementioned advantages of the FPL strategy are conducive to flexible and rapid preparation of high-quality micro/nanostructures on the surface of GO. Because the process contains no assistant operations, such as chemical etching, the properties of the graphene material are retained. As a matter of fact, through modulation of the GO reduction degree and structural design of the rGO surface, we can change the properties of the rGO film, which allows us to further apply the material to optoelectronic applications. For example, the periodic grating structures have obvious optical trapping and grating coupling effects, which enhance the light absorption by ~20% compared with the smooth surface^5^, and the light-active property of rGO also makes it suitable for realizing a photoresponse over a wide spectrum^31–33^. Our devices can realize stable photoresponsivity (*R* ~ 0.7 mA W^−1^) even when exposed to light with low power (0.1 mW). This is numerically comparable to the response of samples obtained by other reduction methods (e.g., chemical and thermal) but is much larger than that of typical photoreduced samples^31–33^. In addition, the different reduction degrees of rGO along and vertical to the grating structures lead to its in-plane effective refractive indexes being different in the two directions, which in turn lays the foundation for the anisotropic response of rGO photodetectors^34^. The conductivity of our devices has an anisotropy ratio of 0.46, which is even larger than that of some natural anisotropic crystals^35,36^. It should be noted that this is the first time that we apply the FPL strategy to the practical application of graphene analogs, not just to the usage of grating structures^25,26^. Our work combines experimental exploration with the in-depth theoretical understanding of high-speed micro/nanopatterning of regular rGO-LIPSS, which not only benefits fundamental physics but also facilitates the practical development of graphene analogs on the industrial scale^37–39^.

## Results

### High-speed femtosecond laser plasmonic patterning of graphene oxide film

**Fig. 1:**
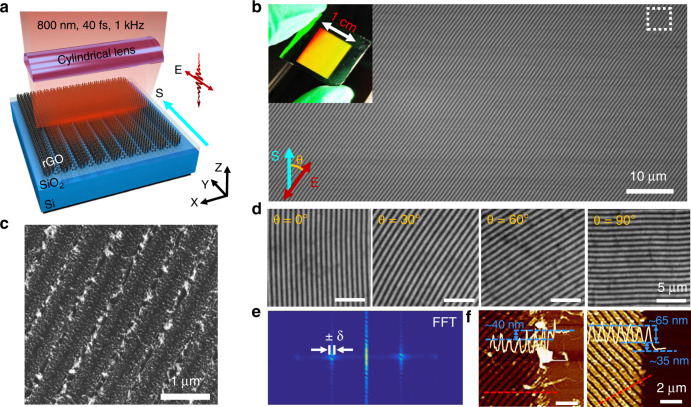
High-speed micro/nanograting processing of GO film using the FPL strategy and morphology characterizations. **a** Schematic of grating processing of a GO film using cylindrical focusing of femtosecond laser pulses. The sample (GO/SiO_2_/Si) is mounted on a three-dimensional (3D) translation stage. The cyan and red arrows represent the directions of the sample scanning (S) and the linear polarization (E) of the laser, respectively. **b** Photograph (insert) and SEM image of the large-area (10 × 12mm^2^) rGO-LIPSS. The uniform structural color (yellow) shows the spatially regular distribution of structures, where *θ* represents the angle between S and E. It can be precisely adjusted by a half-wave plate and a Glan-Taylor prism. **c** High-resolution SEM image of the LIPSS corresponding to the position in the white box in **b**. **d** Observed parallel dependence between the grating orientation and the laser polarization direction. **e** 2D-FFT spectrum suggesting that the period along with its standard deviation (*δ*) is Λ ± *δ* = 680 ± 18nm. **f** AFM images of the rGO-LIPSS on a Si/SiO_2_ substrate. The film thickness is decreased by ~40 nm as a whole, and the depth between the ridges and valleys is ~65 nm

The formation of rGO gratings on a rigid substrate is the basis for further complex operations, which also exhibits potential application in optoelectronics, such as in the fabrication of tuneable terahertz and hyperbolic metamaterial devices^5,7^. Here, GO was prepared using the modified Hummer’s method^40,41^. After being fully dissolved in deionized water, a uniform film with an ~140 nm thickness was spin-coated on the surface of a commercial silicon slice (300 nm SiO_2_). Large-area periodic grating structures were fabricated on the GO film by scanning the fs laser irradiation (Spectra Physics, 40 fs, 800 nm, 1 kHz) through a cylindrical lens (focal length of 50 mm) in ambient air, as shown in Fig. 1a. Because a laser focused by a cylindrical lens is restricted only in one dimension, the focal beam spot on the GO film presents a line profile with measured length and width of ~10 mm and ~12.37 μm, respectively, resulting in a length-to-width aspect ratio as large as approximately 800. The grating formation is determined by tuning the scanning speed and the laser energy fluence within wide ranges of 0.5–200 μm s^−1^ and 25.6–140.2 mJ cm^−2^, respectively. The typical experimental parameters were fixed as 10 μm s^−1^ and 95.6 mJ cm^−2^ here, indicating that the number of spatially overlapping laser pulses is approximately 1200 within the beam spot area or that the irradiation of almost 100 laser pulses accumulates within a 1 μm distance. Details can be seen in the Materials and methods section and Supplementary Information (SI) S1, S2.

Figure 1b presents an example of the grating structure obtained using the aforementioned approach, where a large scanning area with a 10 × 12 mm^2^ coverage and a regular period of ~680 nm can be seen in the photograph (insert) and scanning electron microscopy (SEM, Phenom) images. The detailed topology with plentiful hierarchical nanostructures on the surface is shown in Fig. 1c. In contrast to the traditional laser direct writing method, in which large-area processing often requires multiple scribing of lines, our technology operated here exhibits a much improved efficiency. Using the same parameters in terms of the laser fluence, pulse repetition rate and scanning speed, the time consumption here can be decreased by at least 4 orders of magnitude when texturing a surface area of 10 × 12 mm^2^. In addition, different from the observations of a low-spatial-frequency LIPSS reported on other materials^23–27^, the spatial orientation of our achieved structures is shown to be strictly parallel to the direction of the linear polarization of the incident laser (Fig. 1d). This feature also ensures flexible patterning by changing the scanning direction and laser polarization. However, to obtain higher quality structures, the two directions are recommended to be consistent^42^. Figure 1e shows a typical 2D fast Fourier transform (2D-FFT) spectrum obtained from the SEM image (*θ* = 0°). The calculated structure period is approximately Λ ± *δ* = 680 ± 18 nm, with *δ* being the standard deviation. The regular distribution of the spatial frequency means highly ordered fabrication of the surface structures. Moreover, we found that the period of the structures is slightly less than the laser wavelength and remains almost invariable with the pulse shot number and the energy fluence. This phenomenon may be due to the commonality of carbon materials, which has been reported earlier^27^. For the different processing parameters, the variation tendency of the structure period can be seen in SI S3. The dynamic process of grating structure formation in a large area can be found in Supplementary Movies (SM) SM1, SM2, and SM3.

### Periodic photoreduction of graphene oxide film

Here, along with the micro/nanopatterning process, it should be emphasized that the GO film is simultaneously photoreduced, indicating the generation of an rGO-LIPSS. Because the oxygen-containing functional groups are readily removed from the surface region by both nonthermal (two-photon absorption, for instance) and thermal photoreduction effects, the surface morphology of GO will accordingly change during the laser irradiation^15,43^. Generally, the thickness of the GO film decreases after photoreduction^15^, so a preliminary judgment of the reduction state of the GO sample can be made based on just its thickness comparison before and after laser irradiation.

In our experiment, two phenomena need to be noted: first, in the entire illuminated area, an overall decrease of ~40 nm in the film thickness can be observed. Second, the thickness difference between the ridges and valleys is ~65 nm for the grating structures, with no damage to the silicon substrate during the laser processing, as shown by the atomic force microscopy (AFM, Bruker, Billerica) images in Fig. 1f. For the first phenomenon, we believe that it is the result of the GO surface being reduced as a whole, whereas for the second one, we infer that it is caused by the change in the energy profile of the laser beam spot. Because the deeper morphologies represent the regions of stronger laser–material interaction, the formation of the grating patterns physically indicates the occurrence of a spatially periodic distribution of the laser intensity. In other words, during the scanning (or pulse overlapping) process, the incident laser intensity with a smooth Gaussian distribution on the surface is readily transformed into multiple strong-and-weak energy fringes with a certain period, which results in selective laser interactions with the film. By increasing the laser fluence, we can find that the surface morphology and the depth of the LIPSS change. Although this processing will be accompanied by laser ablation, a higher intensity of the laser is obviously required to reach the threshold^43^. The detailed variation tendency can be seen in SI S4.

**Fig. 2:**
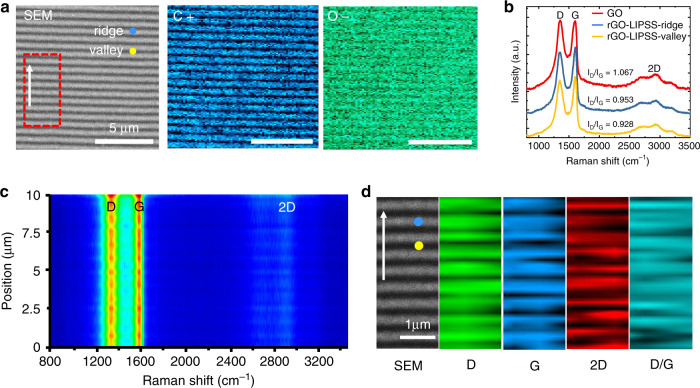
Spatially periodic photoreduction of GO film. **a** SEM image of the rGO-LIPSS and corresponding EDS images of carbon (C+) and oxygen (O−) elements. **b** Comparison of the Raman spectra for the GO film and the ridge and valley positions of the rGO-LIPSS. Both the ridge and valley positions are indicated in **a**. **c** Measured spatial distribution of Raman spectra along the direction (white arrow shown in **a** and **d**) perpendicular to the grating orientation. **d** Measured spatial distributions of three different Raman peaks

This laser-induced periodic photoreduction was more intuitively confirmed by the spatial distribution of the chemical composition measured using energy dispersive spectrometry (EDS, Phenom Prox). Figure 2a shows the content maps of the different elements of carbon (C+) and oxygen (O−) on the LIPSS of the film. Clearly, the spatial distribution patterns of the chemical elements are highly consistent with the grating structures. With the geometric undulations of the rGO-LIPSS, we can find that the elemental constituents are different at the ridge and valley positions/localizations. Here, the grating structure period is approximately Λ = 680 nm, while the structure depth is only approximately *d* = 65 nm, leading to a surface fluctuation with a width-to-depth ratio of approximately 5:1. Such a large aspect ratio can enable us to consider that the surface morphology has no influence on the EDS signal collection; the obtained periodic bright-dark EDS patterns of the rGO-LIPSS are mainly caused by the uneven degree of the photoreduction process. To clarify the above deduction, we also characterized the film by measuring the Raman spectra (Horiba, Jobin Yvon) within a wavenumber range from 800 to 3500 cm^−1^ under excitation by a continuous 532 nm laser. The representative *sp*^2^ vibration of atoms and in-plane disturbance of disordered graphite generally occur in carbon materials, which correspond to the *G* peak at ~1594 cm^−1^ and *D* peak at 1351 cm^−1^, respectively^38^. Another peak occurring at ~2920 cm^−1^, which is the 2D band that indicates the atom stacking morphology of multilayer graphene, can also be observed. The *D*/*G* intensity ratio is widely used to analyse the reduction degree of GO. The smaller this ratio is, the higher the reduction degree.

Figure 2b shows the Raman spectra measured for the GO film without any laser treatment and for the ridge and valley positions/localizations on the rGO-LIPSS. Regardless of the case, three typical peaks (D, G, and 2D) can always be seen, whereas the *D*/*G* intensity ratios are slightly different. Compared to the value of *I*_D_/*I*_G_ = 1.067 for the pristine GO film, the values at the ridge (0.953) and valley (0.928) positions of the rGO-LIPSS decrease, which suggests C–C bond recovery of the material and its gradual graphitization^44,45^. The *I*_D_/*I*_G_ values for different samples and at different locations are the average results from multiple tests (Table S2), and the trend can be clearly illustrated by the spatially resolved Raman intensity distribution of the rGO-LIPSS samples. When the sample was scanned perpendicular to the LIPSS orientation, we found that the variations in the Raman peak intensities well matched the topographic undulations of the rGO-LIPSS (Fig. 2c). Furthermore, when we performed an area-scanning measurement, periodic distributions of the different Raman peaks are evidently shown (Fig. 2d). Therefore, we can conclude that during rGO-LIPSS formation, the GO film is not only photoreduced but also has different degrees of reduction in the ridge and valley regions. To quantitatively analyse the reduction, both X-ray photoelectron spectroscopy (XPS, Thermo Escalab 250Xi) and X-ray diffraction (XRD, Rigaku SmartLab) were employed for the measurement. Detailed descriptions can be found in SI S5.

### Simulation analysis of plasmonic formation on graphene oxide film

**Fig. 3:**
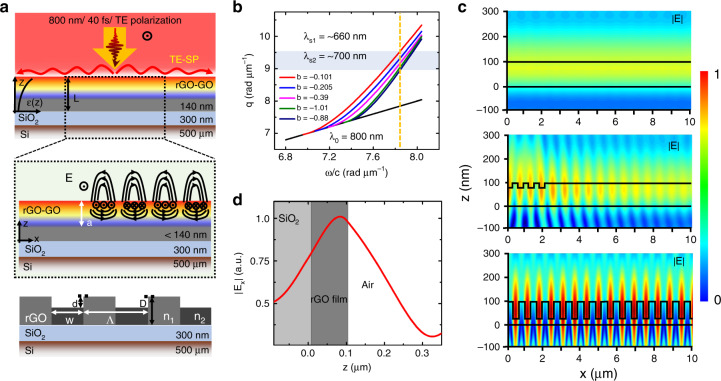
Simulation analysis of the plasmonic formation on GO film. **a** Schematic diagram of rGO-LIPSS formation. Upper: schematic of the TE-SP excitation at the rGO-air interface. The unusual TE-SP wave is excited following the gradient change in the DP distribution (*ε*(*z*)) from the surface to the interior of the rGO/GO sample (along the *z* direction) after femtosecond laser irradiation. “*L*” represents the initial thickness of the GO film before processing. Middle: Detailed description of TE-SP excitation. “*a*” is the thickness of the inhomogeneous rGO film. Bottom: Formation of the rGO-LIPSS after processing. “Λ” is the center-to-center distance between two grooves, and “*w*” and “*d*” represent the width and depth of the grooves, respectively. “*D*” is the final thickness of the rGO film after processing. “*n*_1_” and “*n*_2_” are the refractive indexes at the ridge and valley positions, respectively. **b** Dispersion relation of the TE-SP wave for different inhomogeneity strengths (*b*) and carrier densities (*N*). The dispersion of light in vacuum is shown as the black line. *λ*_*0*_ and *λ*_*s*_ represent the wavelengths of the incident light and TE-SP wave, respectively. **c** Simulated intensity fringes of the *E* field distribution without (upper) and with (middle, bottom) phase matching using the FDTD methods. The depths of the initial and terminal ripples are defined as 20 and 65nm, respectively. **d** Calculated spatial profile of the *E* field intensity for the inhomogeneous film

The formation of the rGO-LIPSS is a complex process including miscellaneous physical mechanisms, whereas we can use a semiphenomenological theoretical model for guidance. The nonthermal or thermal photoreduction effects play a crucial role during the fs laser irradiation of GO film^17^. With the gradual accumulation of laser pulses on the material surface, the degree of reduction can vary from the surface to the interior. This results in an inhomogeneous DP distribution along the depth, whose gradually decreasing values meet the basic conditions for the excitation of the TE-SP wave (Fig. 3a, upper)^28,29^. Consequently, the TE-SP wave is excited and interferes with the incident light to form periodic energy deposition fringes. Then, spatially periodic reduction and ablation of the GO surface occur, and the positive feedback further promotes the excitation of TE-SPs. More importantly, the interference fringe intensity at any point of the film is an integral effect of the incident and scattered light over a large area, so-called nonlocal feedback formation. Due to the high carrier concentrations in the graphene material, the excited TE-SP wave can propagate a long distance, and the feedback is strong enough to regulate the energy deposition (Fig. 3a, middle)^46^. Therefore, when the fs laser spot is scanned in a certain direction, the partially overlapping laser energy distributions will duplicate the existing periodic patterns onto the new laser-exposed positions step by step, resulting in large-area uniform formation of the LIPSS (Fig. 3a, bottom). This kind of nonlocal feedback mechanism presents excellent robustness against disturbances, which makes rGO-LIPSS formation more flexible and extensible^22^. Although grating-like structures have already been fabricated by other methods, such as laser direct writing and beam interference^5,19^, both the efficiency and robustness of our FPL strategy are undoubtedly highlights. More details on the flexible sample processing can be found in SI S6 and S7 and SM S4.

To further elucidate the formation of the rGO-LIPSS, a physical model is introduced as follows: first, we assume that the degree of reduction of the GO film gradually decreases with the material depth (*z* direction in Fig. 3a). Starting from the electromagnetic field theory, the complex amplitude of the electric (*E*) field distribution near the air-rGO interface satisfies the Helmholtz equation. Considering the depth-dependent influence of the incident laser beam on the rGO film, *ε*(*z*) is defined as the *z*-dependent DP and can be derived from the carrier density distribution (*N*) using the Drude model. After introducing *ε*(*z*) into the Helmholtz equation, the *E* field in the rGO film can be solved. Considering the continuity boundary condition at the air-rGO interface, the dispersion relation of the TE-SP wave propagating along the rGO surface should satisfy the following equation:

1 $$\left( {q_{\mathrm{s}}a} \right)^2 = \left( {\frac{{\omega a}}{c}} \right)^2 + \left[ {\frac{1}{2} + \frac{1}{{K_\rho \left( s \right)}}\left. {\frac{{\partial K_\rho \left( {{\mathrm{su}}} \right)}}{{\partial u}}} \right|_{u = 1}} \right]^2$$

where *q*_s_ is the wavevector of the TE-SP wave and *c* and *ω* are the speed and frequency of incident light, respectively. *K*_*ρ*_(su) is the Bessel function of the second kind, which simplifies the calculation of the Helmholtz equation^28^.

Here, $u = 1 + \frac{{D - z}}{a}$, $s^2 = \left( {q_{\mathrm{s}}a} \right)^2 - \,\varepsilon _{\mathrm{L}}\left( {\frac{{\omega a}}{c}} \right)^2 + \,\varepsilon _{\mathrm{L}}\left( {\frac{{\omega _{\mathrm{pa}}}}{c}} \right)^2\left( {1 - \frac{1}{b}} \right)$, $\rho ^2 = \frac{{\varepsilon _{\mathrm{L}}}}{b}\left( {\frac{{\omega _{\mathrm{p}}a}}{c}} \right)^2 + \frac{1}{4}$, and $\omega _{\mathrm{p}}^2 = \frac{{4\pi e^2N_0}}{{\varepsilon _{\mathrm{L}}m_{{\mathrm{eff}}}}}$. *ω*_p_ is defined as the inherent plasma frequency of bulk rGO material associated with carrier density *N*_0_ at the air-rGO interface (*z* = *D*), and *m*_eff_ is the carrier effective mass. *ε*_L_ is the frequency-independent part of the DP of the film. *a* (>0) is the thickness of the inhomogeneous rGO layer, and the dimensionless parameter *b* describes the strength of the inhomogeneity. Subsequent studies found that the specific form of the *z*-dependent gradient of the carrier density in the rGO film has little influence on the results of the *E* field distribution. Detailed information can be found in SI S8.

Dispersion relation curves were obtained, as shown in Fig. 3b, which illustrates the possibility of the generation of a collective oscillation of pseudo-surface currents. The values of *N*_0_ and *b* satisfying the TE-SP wave excitation have a wide range. They are mainly limited by the DP variation of the GO film, the change range of the plasma frequency (*ω* > *ω*_p_), and the wavelength of the TE-SP wave in the measurements (*q*_s_ ~ 8.98–9.52 rad μm^−1^). Other relevant parameters are shown in Tables S1 and SI S9.

To support the experimental data, simulations were carried out to determine the *E* field distribution using the finite difference time domain (FDTD) method. By introducing four equally spaced grooves as a phase matching excitation, coupling of incident light into the TE-SP wave excitation can be efficiently realized, as compared in Fig. 3c (upper and middle)^23,27^. The TE-SP wave propagates on the surface of the inhomogeneous film and further induces grating structures. As the grating depth increases, the excitation efficiency of the TE-SP wave is improved, and the spatial regularity of the *E* field is also increased (Fig. 3c bottom). Figure 3d shows the spatial profile of the *E* field intensity for the TE-SP wave across the film, wherein the *E* field value extends from the air into the interior of the rGO film, with the maximum intensity occurring at the air–rGO interface.

Here, there are still several problems that need to be further emphasized. First, the *z*-dependent gradient distribution of the DP plays an essential role in the structure formation. Materials with uniform DP distribution are not conducive to coupling of incident light into the TE-SP wave. Second, the thickness of the materials has a great influence on the coupling and interference of incident light with the TE-SP wave. In the experiment, when the thickness of the GO film is less than 50 nm, both the repeatability and regularity of the rGO-LIPSS greatly decrease, which may be due to the decrease in both TE-SP wave generation and the coupling efficiency at the reduced thickness. This may be the reason for the irregular LIPSS formation on other few-layered 2D materials^47,48^. Third, TE-SP wave excitation occurs in the transient nonequilibrium state of the material. The actual optical parameters at the air-rGO interface should be defined as a volume fraction considering both the values of the material and air^23^. The influence of the uniform DP distribution, rGO film thickness and incident laser light on the electric field distribution can be seen in SI S10–S12, respectively.

### On-chip anisotropic photoelectric device fabrication

With the help of the grating coupling effect of the LIPSS and its optical trapping caused by the plentiful hierarchical micro/nanostructures on the surface, our structured rGO samples exhibit the characteristics of relatively high light absorption compared with the smooth film^5,49^. This enhanced optical absorption causes an increase in the temperature of the samples, which in turn promotes the photoelectric conversion due to the excellent photothermoelectric and bolometric properties of graphene^50^. Meanwhile, considering that the different reduction states along the parallel and vertical directions of the LIPSS may result in differentiated photoresponses, it is evident that the structured rGO samples are a promising material for anisotropic photoelectric devices^34–36^.

**Fig. 4:**
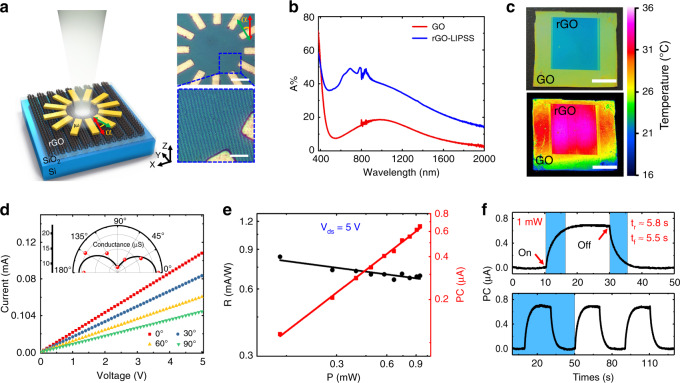
Photoelectric response of the rGO samples with the rGO-LIPSS. **a** Schematic diagram (left) and photographs (right) of the angle-resolved photoelectric measurement of rGO samples. The top-down materials of the samples are gold electrodes (50nm), rGO-LIPSS (~100nm), SiO_2_ film (300nm) and Si substrate. *α* represents the angle between the alignment of the electrode pair used for measurement (the green arrow) and the reference position (the red arrow). Here, there is a deviation of ~10° between the processing direction of the LIPSS and the ideal case. The scale bars are 20 μm (top) and 5 μm (bottom). **b**, **c** Light absorption and corresponding infrared images at the positions of the GO film and the rGO-LIPSS. The scale bars in **c** are 500 μm. **d** α-dependent I–V curves (main) and conductance (inset) of rGO samples. **e** Photoresponsivity (R) and photocurrent (PC) of rGO samples under irradiation by LED white light (OPTO SIGMA, SLA-100). **f** Temporal photoresponse of rGO samples under 1mW irradiation

The schematic architecture of an on-chip photodetector is shown in Fig. 4a. Twelve electrodes (50 nm gold) spaced at an angle of 30° were fabricated on the rGO film. The direction parallel to the LIPSS orientation is marked as a reference (the red arrow), and its anti-clockwise angle with the alignment direction (the green arrow) of the oppositely placed electrode pairs is marked as *α*. Each pair of electrodes was used as the source and the drain. Figure 4b presents a comparison of the light absorption of GO films before and after the laser processing. Within a wide spectral range from the visible to infrared (380–2200 nm), the structured rGO film surface exhibits light absorption with an enhancement of ~20%. The corresponding infrared image shows that the temperature in the laser processed area increases by more than 10 °C (Fig. 4c). As a comparison, the temperature response of a thermally reduced sample was also investigated. The results show that the change in temperature is not significant if the reduction of GO only sample is considered (<1 °C), which further confirms that the surface morphology of the material is more critical. The more regular the LIPSSs are, the greater the change in temperature. A detailed description can be found in SI S13.

Figure 4d shows the electrical properties of the rGO sample, and a typical linear current–voltage (I–V) curve with strong anisotropy can be observed for different electrode connections. The measured maximum and minimum conductivities, corresponding to 21.7 and 8.1 μS, appear along the directions parallel and perpendicular to the LIPSS orientation, respectively, which results in an anisotropy ratio of approximately 0.46. A larger value of this parameter indicates better unidirectional current continuity of the material^35^. Compared to some natural anisotropic crystals, the anisotropy ratio of our artificial film prepared here has a strong competitive advantage^35,36^. Furthermore, we selected the direction with maximal conductivity as a reference and measured the response under irradiation by an LED white light source (OPTO SIGMA, SLA-100). With increasing incident light intensity, the variation in the photocurrent of the rGO sample and its corresponding photoresponsivity (*R*) are shown in Fig. 4e. *R* is almost invariable at ~0.7 mA W^−1^, which is numerically comparable to the response of samples obtained by other reduction methods (e.g., chemical and thermal) and much larger than that of typical photoreduced samples^31–33^. However, the photoresponse speed of our samples is relatively slow, as shown by the temporal measurement in Fig. 4f. The rise and fall times are 5.8 s and 5.5 s, respectively. The reason is that the rGO film surface contains plentiful oxygen-containing functional groups and micro/nanostructures that greatly increase the specific surface area, which hinders the electrical conductivity. The bolometric and photothermoelectric effects, rather than the photovoltaic effect, may play key roles here.

## Discussion

It is of great practical significance to use the FPL strategy to realize high-speed, large-area, and uniform micro/nanofabrication on the surface of 2D materials, whereas both the theoretical and experimental explorations of this method are still in their infancy. Compared with existing femtosecond laser processing technologies, such as direct writing and beam interference, the FPL strategy is mainly based on incident light-SP interference and exhibits nonlinear optical characteristics, so it has superiorities of higher efficiency and stronger robustness against a range of perturbations. Moreover, notably, it should present potential for surface processing with flexible controllability and high accuracy. The correspondence between the LIPSS orientation and the polarization direction of the incident light increases the degrees of freedom for us to control the morphology formation. By adjusting the time delay between laser pulse trains, we can further manipulate the laser processing to achieve more complex topography^51^. The FPL strategy has also been proven to be capable of breaking the diffraction limit by applying suitable experimental parameters^21,47^. Apart from this, we should also emphasize that the phenomenon here is different from others, although similar works have been covered in previous reports^23–27^. The spatial orientation of the grating is parallel rather than perpendicular to the polarization direction of the incident light, leading to a completely different analysis of the laser-matter interaction. This lays the foundation for us to enrich the physical mechanisms of and to fully understand the FPL processing procedure.

In addition, we should also note that the design and processing of the micro/nanostructures on the surface of the sample are the basis of the early stage, whose final purpose is to optimize the properties of materials for further application. Through the characterizations and photoelectric measurements of the rGO-LIPSS, the samples processed by our FPL strategy are proven to retain most of the properties of graphene and become even better in some aspects, such as higher light absorption and an anisotropic photoresponse. Therefore, we should fully use our technology for the rapid, high-quality processing of micro/nanostructures and make full use of the advantages of the sample in terms of its unique material and structure properties. By combining the FPL strategy with other technologies, we believe that the properties of the rGO-LIPSS can be further optimized and the related applications can be further expanded.

In conclusion, by adopting the FPL strategy, we have demonstrated high-speed, large-area, and uniform fabrication of micro/nanograting structures on GO films with the simultaneous occurrence of in situ photoreduction. The rGO-LIPSS exhibited anomalous features, including a spatial arrangement parallel to the direction of the laser polarization and evident robustness against perturbations. To elucidate the underlying physics of the structure formation, a semiphenomenological model combined with the electric field distribution was applied for guidance. By introducing the concept of an inhomogeneous dielectric permittivity layer in the GO material, we simulated the TE-SP excitation and its subsequent interference with the incident light to form periodic energy deposition parallel to the laser polarization. Owing to the in-plane unidirectional arrangement of the rGO-LIPSS and hierarchical micro/nanostructures on the surface, an on-chip device with an obvious anisotropic photoresponse was developed. Our work, for the first time, provides an example of the practical usage of the FPL strategy in the processing of 2D graphene analogues and the implementation of related micro/nanodevices, which demonstrates the great potential in the field of high-speed and precise manufacturing.

## Materials and methods

### Materials

The GO used in the experiment was prepared by the modified Hummer method and then dissolved and broken in deionized water to form a uniform GO solution (40 mg ml^−1^). After surface hydrophilic treatment of a silicon wafer (300 nm SiO_2_) using oxygen plasma, the GO solution was spin-coated (2500 r and 30 s) on it to realize a thin film. The wafer was then kept on a hot plate at 55 °C for 1 min, and a film with a uniform thickness of 10 nm was obtained. By repeating the spin-coating process, we could precisely control the thickness.

### Femtosecond laser fabrication

rGO-LIPSSs were fast patterned using a commercial chirped-pulse-amplification fs-laser system (Spitfire Ace, Spectra Physics). The central wavelength, repetition rate, and pulse width were 800 nm, 1 kHz, and 40 fs, respectively. The maximum energy of each laser pulse was ~7 mJ. The laser beam was focused through a cylindrical lens, forming a line-shaped focal spot with a length-to-width aspect ratio of approximately 800. A half-wave plate and a Glan-Taylor prism were used to vary the pulse energy and the polarization direction of the laser. Here, the typical scanning speed, laser power and overlapping pulse number were tuned to 10 μm s^−1^, 95.6 mJ cm^−2^, and 1200, respectively. The real-time rGO-LIPSS processing was directly monitored with a homemade microimaging system (MIS), as shown in SM2 and SM3. In the MIS system, 10× and 100× objective lenses were used for observation.

### Characterization

Optical images were captured by a confocal microscope (Keyence, VK-X1000). Characterizations such as SEM (Phenom, Eindhoven, Netherlands) and AFM (Bruker, Billerica, MA, USA) were performed to examine the detailed morphologies. The chemical compositions of the rGO film were analysed by XPS (Thermo Escalab 250Xi) and XRD (Rigaku SmartLab). The spatial element distribution was analysed using EDS (Phenom, Prox). The Raman spectra were measured by a spectrometer (LabRAM HR Evolution) with an excitation wavelength of 532 nm. The light absorption of the GO film and rGO-LIPSS was investigated by a UV–Vis–NIR spectrometer (Agilent, Cary 5000) over a wavelength range of 200–2000 nm. The rGO samples were illuminated by an LED light source (OPTO SIGMA, SLA-100), and the photoresponse was measured using a 4200A-SCS Parameter Analyzer (Keithley, USA). The same LED light with a power of 65 mW and an infrared camera (Image@IR 8300) were used to characterize the photothermal properties and record the temperature.

### Theoretical simulation

The formation of the rGO-LIPSS was theoretically simulated by the finite difference time domain (FDTD) method (Lumerical FDTD solutions software package). A rectangular box with dimensions of 400 nm × 10 μm was fixed as the simulation region. The top and bottom boundaries of the region were set as perfectly matched layers, while periodic boundary conditions were applied to the left and right boundaries. The light source was a TE-polarized plane wave with a Gaussian temporal profile, which was normally incident on the rGO surface from top to bottom. Its electric field vector was parallel to the LIPSS. The *E* field distribution of the rGO structure was obtained by Fourier transformation of the time–domain *E* field at a wavelength (*λ*_0_) of 800 nm. The thickness of the rGO film (*D*) was 100 nm. The width (*w*) and depth (*d*) of the grooves were fixed as 250 and 65 nm, respectively. The center-to-center distance (Λ) between two grooves was fixed as 650 nm.

## Supplementary information

Supplementary Information

Video of rGO-LIPSS processing-1

Video of rGO-LIPSS processing-2

Video of rGO-LIPSS processing-3

Observation of uniform large-area rGO-LIPSS

## References

[1] Georgakilas V (2016). Noncovalent functionalization of graphene and graphene oxide for energy materials, biosensing, catalytic, and biomedical applications. Chem. Rev..

[2] Compton OC, Nguyen ST (2010). Graphene oxide, highly reduced graphene oxide, and graphene: versatile building blocks for carbon-based materials. Small.

[3] Patchkovskii S (2005). Graphene nanostructures as tunable storage media for molecular hydrogen. Proc. Natl Acad. Sci. USA.

[4] Han N (2013). Improved heat dissipation in gallium nitride light-emitting diodes with embedded graphene oxide pattern. Nat. Commun..

[5] Lin H (2019). A 90-nm-thick graphene metamaterial for strong and extremely broadband absorption of unpolarized light. Nat. Photonics.

[6] Yan HG (2013). Damping pathways of mid-infrared plasmons in graphene nanostructures. Nat. Photonics.

[7] Ju L (2011). Graphene plasmonics for tunable terahertz metamaterials. Nat. Nanotechnol..

[8] Gomez-Diaz JS, Tymchenko M, Alù A (2015). Hyperbolic metasurfaces: surface plasmons, light-matter interactions, and physical implementation using graphene strips [Invited]. Optical Mater. Express.

[9] Liu ZM (2017). Three-dimensional self-organization in nanocomposite layered systems by ultrafast laser pulses. ACS Nano.

[10] Guo LJ (2007). Nanoimprint lithography: methods and material requirements. Adv. Mater..

[11] Tokel O (2017). In-chip microstructures and photonic devices fabricated by nonlinear laser lithography deep inside silicon. Nat. Photonics.

[12] Zheng XR (2019). Patterning metal contacts on monolayer MoS_2_ with vanishing Schottky barriers using thermal nanolithography. Nat. Electron..

[13] Garcia R, Knoll AW, Riedo E (2014). Advanced scanning probe lithography. Nat. Nanotechnol..

[14] Vorobyev AY, Guo CL (2013). Direct femtosecond laser surface nano/microstructuring and its applications. Laser Photonics Rev..

[15] Zhang YL (2010). Direct imprinting of microcircuits on graphene oxides film by femtosecond laser reduction. Nano Today.

[16] Zheng XR (2015). Highly efficient and ultra-broadband graphene oxide ultrathin lenses with three-dimensional subwavelength focusing. Nat. Commun..

[17] Gengler RYN (2013). Revealing the ultrafast process behind the photoreduction of graphene oxide. Nat. Commun..

[18] Strong V (2012). Patterning and electronic tuning of laser scribed graphene for flexible all-carbon devices. ACS Nano.

[19] Guo L (2012). Two-beam-laser interference mediated reduction, patterning and nanostructuring of graphene oxide for the production of a flexible humidity sensing device. Carbon.

[20] Birnbaum M (1965). Semiconductor surface damage produced by ruby lasers. J. Appl. Phys..

[21] Bonse J (2017). Laser-induced periodic surface structures—a scientific evergreen. IEEE J. Sel. Top. Quantum Electron..

[22] Öktem B (2013). Nonlinear laser lithography for indefinitely large-area nanostructuring with femtosecond pulses. Nat. Photonics.

[23] Wang L (2017). Plasmonic nano-printing: large-area nanoscale energy deposition for efficient surface texturing. Light Sci. Appl..

[24] Huang J (2019). Cylindrically focused nonablative femtosecond laser processing of long-range uniform periodic surface structures with tunable diffraction efficiency. Adv. Opt. Mater..

[25] Huang J (2019). Fabrication of highly homogeneous and controllable nanogratings on silicon via chemical etching-assisted femtosecond laser modification. Nanophotonics.

[26] Sidhu MS, Munjal P, Singh KP (2018). High-fidelity large area nano-patterning of silicon with femtosecond light sheet. Appl. Phys. A.

[27] Huang M (2009). Origin of laser-induced near-subwavelength ripples: interference between surface plasmons and incident laser. ACS Nano.

[28] Shvartzburg A, Petite G, Auby N (1999). *S*-polarized surface electromagnetic waves in inhomogeneous media: exactly solvable models. J. Opt. Soc. Am. B.

[29] Kim K (2008). Excitation of s-polarized surface electromagnetic waves in inhomogeneous dielectric media. Opt. Express.

[30] Sun ZJ (2014). Artificial TE-mode surface waves at metal surfaces mimicking surface plasmons. Opt. Express.

[31] Chitara B (2011). Infrared photodetectors based on reduced graphene oxide and graphene nanoribbons. Adv. Mater..

[32] Cao Y (2017). Fully suspended reduced graphene oxide photodetector with annealing temperature-dependent broad spectral binary photoresponses. ACS Photonics.

[33] Yao HB (2010). Direct fabrication of photoconductive patterns on LBL assembled graphene oxide/PDDA/titania hybrid films by photothermal and photocatalytic reduction. J. Mater. Chem..

[34] Flanders DC (1983). Submicrometer periodicity gratings as artificial anisotropic dielectrics. Appl. Phys. Lett..

[35] Xin W (2018). Black-phosphorus-based orientation-induced diodes. Adv. Mater..

[36] Island JO (2015). TiS_3_ transistors with tailored morphology and electrical properties. Adv. Mater..

[37] You R (2019). Laser fabrication of graphene-based flexible electronics. Adv. Mater..

[38] Jiang HB (2020). Review of photoreduction and synchronous patterning of graphene oxide toward advanced applications. J. Mater. Sci..

[39] Zhang YL (2020). A “Yin”-“Yang” complementarity strategy for design and fabrication of dual-responsive bimorph actuators. Nano Energy.

[40] Hummers WS, Offeman RE (1958). Preparation of graphitic oxide. J. Am. Chem. Soc..

[41] Jiang WS (2017). Reduced graphene oxide-based optical sensor for detecting specific protein. Sens. Actuators B.

[42] Gnilitskyi I (2017). High-speed manufacturing of highly regular femtosecond laser-induced periodic surface structures: physical origin of regularity. Sci. Rep..

[43] Kymakis E (2013). Flexible organic photovoltaic cells with in situ nonthermal photoreduction of spin-coated graphene oxide electrodes. Adv. Funct. Mater..

[44] Tian H (2014). Cost-effective, transfer-free, flexible resistive random access memory using laser-scribed reduced graphene oxide patterning technology. Nano Lett..

[45] Lin J (2014). Laser-induced porous graphene films from commercial polymers. Nat. Commun..

[46] Gao WL (2012). Excitation of plasmonic waves in graphene by guided-mode resonances. ACS Nano.

[47] Beltaos A (2014). Femtosecond laser induced periodic surface structures on multi-layer graphene. J. Appl. Phys..

[48] Kasischke M (2018). Simultaneous nanopatterning and reduction of graphene oxide by femtosecond laser pulses. Appl. Surf. Sci..

[49] Ren HY (2017). Hierarchical graphene foam for efficient omnidirectional solar-thermal energy conversion. Adv. Mater..

[50] Xie C (2017). Photodetectors based on two-dimensional layered materials beyond graphene. Adv. Funct. Mater..

[51] Jalil SA (2019). Formation of controllable 1D and 2D periodic surface structures on cobalt by femtosecond double pulse laser irradiation. Appl. Phys. Lett..

