# Supplementary material for: High-speed femtosecond laser plasmonic lithography and reduction of graphene oxide for anisotropic photoresponse
Source: Light Sci Appl. 2020 Apr 26;9:69. doi: 10.1038/s41377-020-0311-2 (PMC7183510; doi:10.1038/s41377-020-0311-2)
Supplement: Supplementary file 1 — Supplementary Information [file 41377_2020_311_MOESM1_ESM.docx]

**Supplementary Information for**

**High-speed femtosecond laser plasmonic lithography and reduction of graphene oxide for anisotropic photoresponse**

Tingting Zou^1,2^, Bo Zhao^1,3^, Wei Xin^1^*, Ye Wang^1,2^, Bin Wang^1,2^, Xin Zheng^1,2^, Hongbo Xie^1,2^, Zhiyu Zhang^4^, Jianjun Yang^1^* and Chun-lei Guo^1,5^*

^1^ State Key Laboratory of Applied Optics, Changchun Institute of Optics, Fine Mechanics and Physics, Chinese Academy of Sciences, Changchun, 130033, China. E-mail: [weixin@ciomp.ac.cn](mailto:weixin@ciomp.ac.cn); jjyang@ciomp.ac.cn.

^2^ Center of Materials Science and Optoelectronics Engineering, University of Chinese Academy of Sciences, Beijing, 100049, China.

^3^Department of Electronic Information and Physics, Changzhi University, Changzhi 046011, China.

^4^Key Laboratory of Optical System Advanced Manufacturing Technology, Changchun Institute of Optics, Fine Mechanics and Physics (CIOMP), Chinese Academy of Sciences (CAS), Changchun 130033, China.

^5^The Institute of Optics, University of Rochester, Rochester, New York, 14627, United States. E-mail: [chunlei.guo@rochester.edu](mailto:chunlei.guo@rochester.edu)

**S1. Description of the incident laser parameters**

In the experiment, both the laser fluence and the number of pulse overlapping are key factors influencing the rGO-LIPSS formation. Here, a cylindrical lens with a focal length of 50 mm was employed for the laser focusing, so that the focal beam spot became compressed only in one direction. The GO film was placed at 100 μm away before the focus, as shown in Figure S1a. The spatial distribution of the laser intensity on the sample surface was simulated using the Zemax software. Following the definition of the *x*, *y*, and *z* directions in the main text, we assumed that the focal laser spot has a Gaussian intensity distribution along the *x* direction (with a large size of *l_f_ =* 10 mm) and a relatively uniform distribution in the *y* direction (with a small size of *d_f_ =* 12.37 μm), as shown in Figure S1b.

In addition, to study the influence of the pulse overlapping on the generation of rGO-LIPSS, we made calculations using a formula *N = f × d_f_ /v*, where the repetition rate *f =* 1 kHz, and the laser scanning speed *v* varying from 0.5 to 200 μm s^-1^. Correspondingly, the number of pulse overlapping is located within a range of *N* = 62~24740.

**
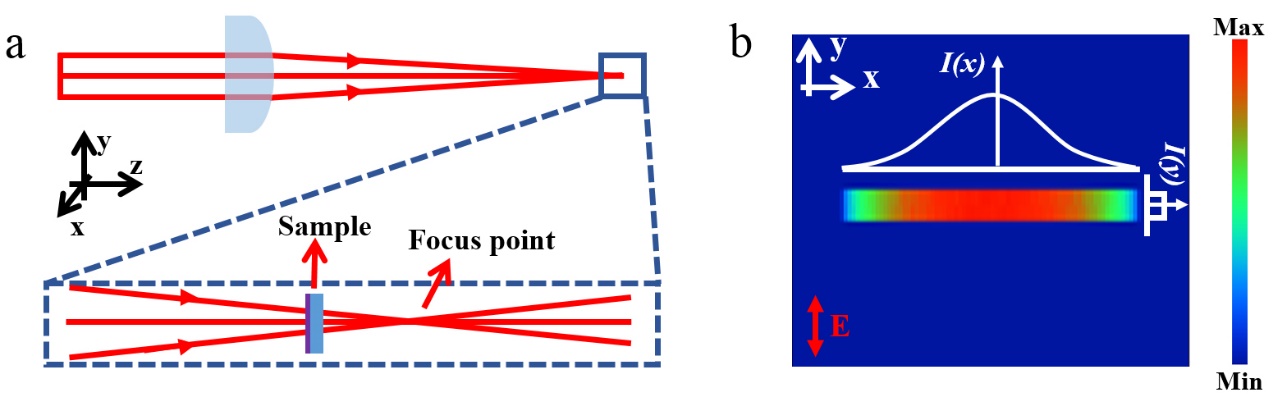
**

**Figure S1.** **a** Schematic diagram of the laser beam focusing by a cylindrical lens. **b** The spatial distribution of the focal laser beam intensity simulated by Zemax.

**S2. Dynamic ranges of femtosecond laser processing**

Based on the above calculations and the experiments, we successfully determined the suitable conditions for the rGO-LIPSS formation with different film thicknesses in terms of the laser fluence (*F*) and the number of pulse overlapping (*N_p_*), as shown in Figure S2a-2f. Clearly, the window condition for generating rGO-LIPSS is strongly dependent on the thickness of the GO film. For example, when the film thickness is between 100 nm and 200 nm, the LIPSS is preferable to get within a large dynamic range. However, if the film thickness is either less than 50 nm or large than 300 nm, the generation of rGO-LIPSS becomes more difficult.

**
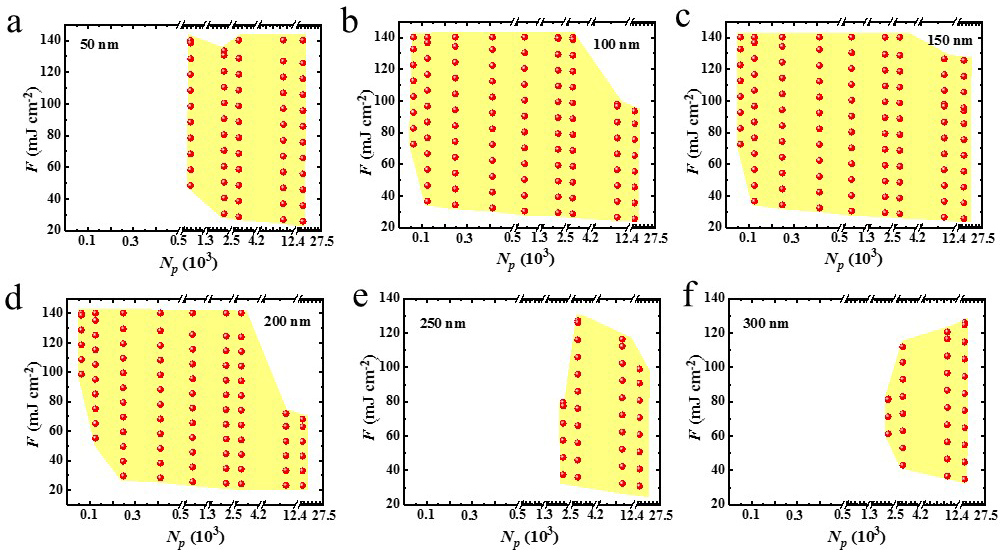
**

**Figure S2.** Obtained window conditions for the formation of rGO-LIPSS in terms of the laser fluence and number of pulse overlapping, when the GO film has several different thicknesses of 50 nm (**a**), 100 nm (**b**), 150 nm (**c**), 200 nm (**d**), 250 nm (**e**) and 300 nm (**f**).

**S3. Variation tendency of the rGO-LIPSS period**

The spatial period Λ of rGO-LIPSS, can be further analyzed from the 2D-FFT information of the SEM image, and its variations with different laser parameters are shown in Figure S3. The thickness of GO film is 140 nm. It is seen that no matter changing the laser fluence or the number of pulse overlapping, the LIPSS period keeps almost constant (Λ = 680 ± 18 nm). This phenomenon may be the common for carbon materials, which has been reported earlier^1^.

**
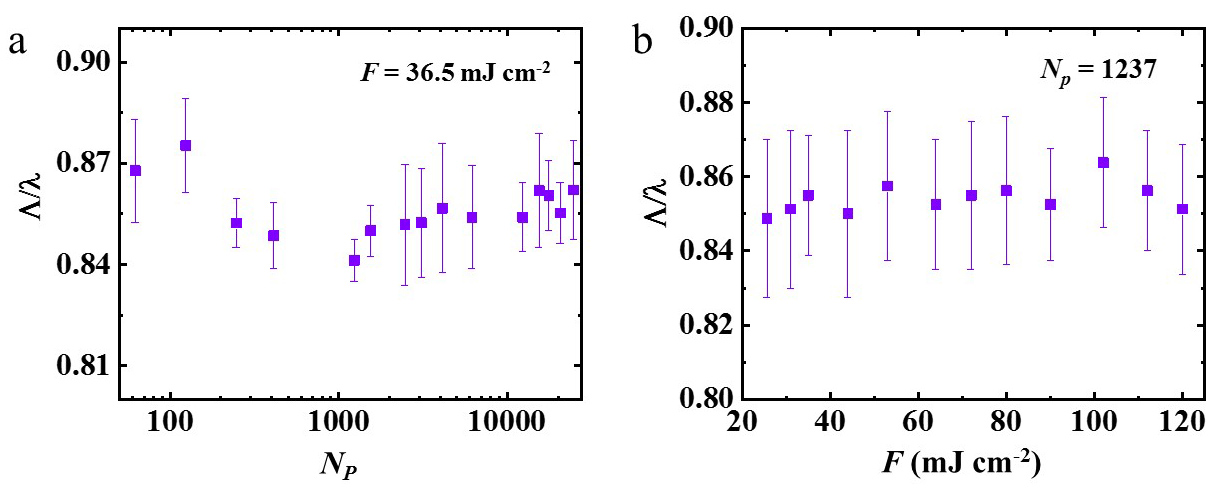
**

**Figure S3.** Measured Λ/λ ratios as a function of different laser parameters. **a** The number of pulse overlapping at the laser fluence of *F* = 36.5 mJ cm^-2^. **b** The laser fluence at the number of pulse overlapping of *N_p_* = 1237.

**S4. Variation tendency of the rGO-LIPSS depth**

Figure S4 presents variations of the measured surface topography of rGO-LIPSS for several different laser fluences. From the statistical analysis of the measurement data, we can see that the modulation depth of the rGO-LIPSS tends to increase gradually for the laser fluences smaller than 23.76 mJ cm^-2^, and then decrease with the larger laser fluences due to the serious ablation process.


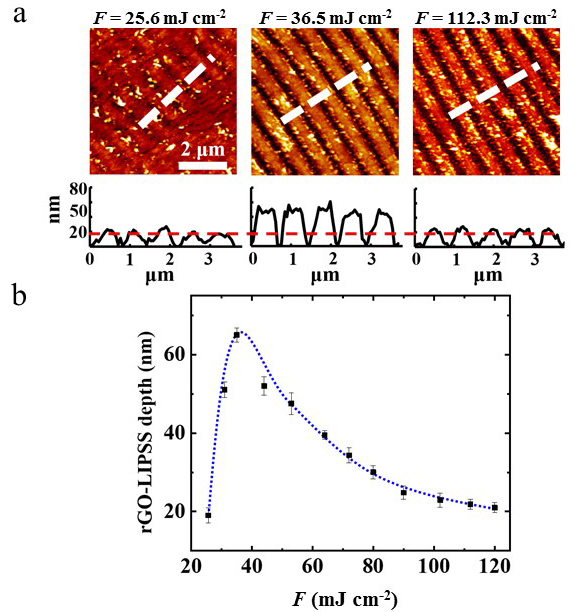


**Figure S4. a** AFM images of the rGO-LIPSS obtained with different laser fluences. **b** Variation of the modulation depth of the rGO-LIPSS with increasing laser fluence.

**S5. Analysis of GO and rGO-LIPSS with XRD and XPS methods**

To quantitatively analyze the reduction degree of the GO film, both X-ray photoelectron spectroscopy (XPS, Thermo escalab 250Xi) and X-ray diffraction (XRD, Rigaku SmartLab) were employed. In the XRD measurement of the GO film, there is a characteristic diffraction peak at 2θ = 10.4°. However, it becomes disappeared for the rGO-LIPSS and a new diffraction peak begins to occur at 2θ = 21°, which indicates oxygen reduction of the GO film (Figure S5a).

From the XPS results shown in Figure S5b, we found that the intensity of O_1s_ peaks at the region of rGO-LIPSS is significantly weaker than that at GO film. In addition, as shown in Figure S5c, after the femtosecond laser treatment, the area ratios of both characteristic peaks of C-O (287.5 eV) and C=O (289.0 eV) are 21.3% and 1.5%, respectively, which are lower than that of GO film (22.9% and 8.6% for C-O and C=O, respectively). However, the *sp^2^* hybrid peak intensity at 284.8 eV (C=C) increase remarkably, and the ratios of GO film and rGO-LIPSS are 68.5% and 77.2%, respectively. All these results suggest that the GO film has been reduced after laser irradiation due to the removal of oxygen functional groups.


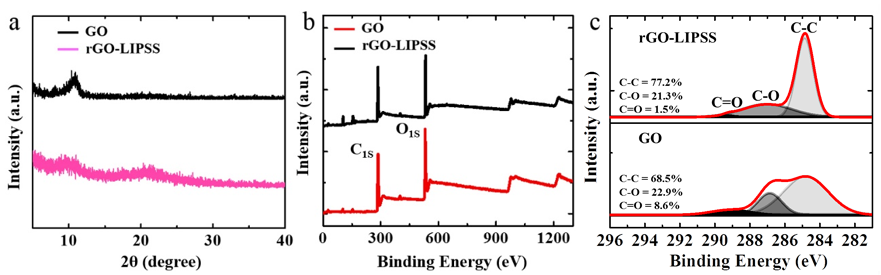


**Figure S5.** Measured XRD (**a**) and XPS (**b**, **c**) results for both the GO film and rGO- LIPSS.

**S6. Robustness of the LIPSS formation against perturbations**

The robustness against perturbations on the rGO-LIPSS formation is shown in Figure S7a and S6b which taken from **SM4**. Thanks to the unique feedback mechanisms in the nonlinear systems, the femtosecond laser processing exhibits excellent robustness against a range of perturbations^2^. It can be seen that even if the defect size is comparable to the incident light wavelength, the effect of diffraction on the formation of rGO-LIPSS is also negligible.


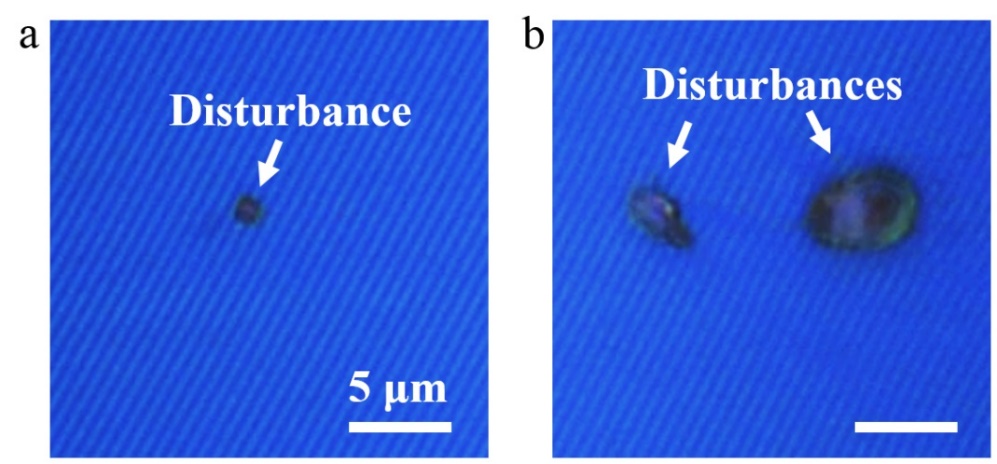


**Figure S6.** Microscope images of the rGO-LIPSS formation with strong robustness against distinct perturbations. (**a**) For the defect size less than 1.2 μm. (**b**) For the defect sizes larger than 2.5 μm. All the scale bars are 5 μm.

**S7. Flexible processing of rGO-LIPSS**

The processing of rGO-LIPSS is flexible and controllable. Figures S7a and S7b show the rGO-LIPSS formation on a silicon and a quartz substrate, respectively. With the help of a macro-scale template, a so-called “GPL” pattern of rGO-LIPSS can be fast imprinted on the GO film by only one-direction scanning of femtosecond laser pulse. Since each character contains about 6000 regular grating structures inside, each pattern (5 × 4 mm^2^) is seen colorful by the naked eyes. More interestingly, the produced rGO-LIPSSs are so robust that it can be completely transferred to the surface of other materials. Figure S7c shows an example of structure transferring onto the SiO_2_ substrate, which is also evidenced by the structural colors. Of course, this method can be extended to the structure transfer onto a flexible Polydimethylsiloxane (PDMS) substrate (Figure S7d).


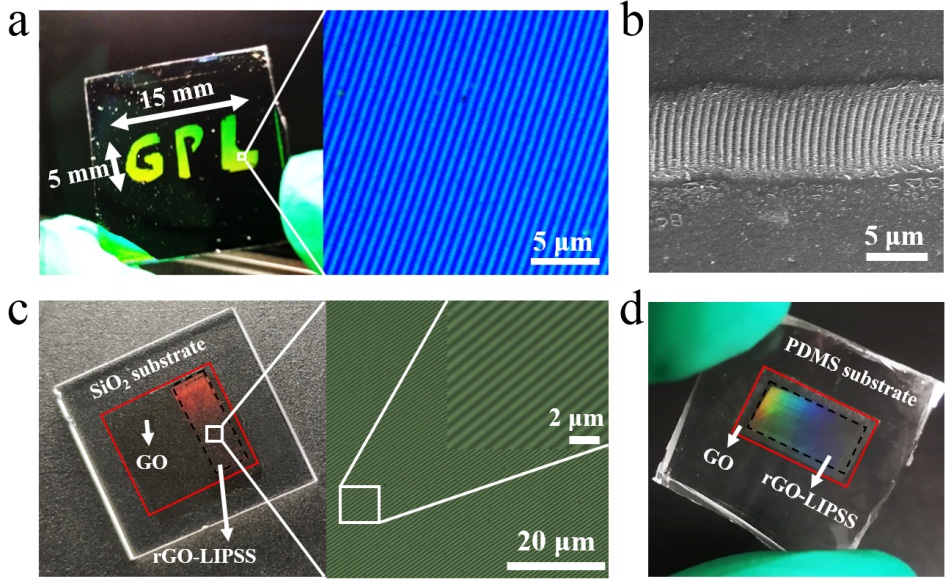


**Figure S7.** Formation of rGO-LIPSS on a Si (**a**, photograph) and a SiO_2_ (**b**, SEM image) substrate, respectively. Examples of the rGO-LIPSS transfer onto a SiO_2_ (**c**) and a PDMS (**d**) substrate.**S8. Simulation of the plasmonic lithography process**

We assume that the degree of oxygen reduction of the GO film gradually decreases with the material depth, leading to a *z*-dependent dielectric permittivity (DP, *ε* (*z*)) in the rGO film. From the Drude model, the DP can de deduced from the carrier’s density distribution (*N*), and the *z*-dependent *N* is given by^3^:

$N\left(z\right)=N_{0}\left[1-\frac{1}{b}+\frac{F^{2}\left(z\right)}{b}\right], F\left(z\right)=\left(1+\frac{D-z}{a}\right)^{-1}$ (1)

where *N_0_* is the carrier’s density at the surface (*z* = *D*) of the rGO film irradiated by femtosecond laser pulses, the dimensionless parameter *b* describes the inhomogeneity strength, *a* (>0) represents the thickness of the inhomogeneous rGO layer. Here, the application of function *F* (*z*) is to introduce a *z*-dependent gradient of the carrier density in the rGO film. Based on the carrier distribution, the dielectric constant can be deduced as follows:

$\varepsilon\left(z\right)=\varepsilon_{L}\left\{1-\frac{\omega_{p}^{2}}{\omega^{2}}\left[1-\frac{1}{b}+\frac{F^{2}\left(z\right)}{b}\right]\right\},$ $\omega_{p}^{2}=\frac{4\pi e^{2}N_{0}}{\varepsilon_{L}m_{eff}}$ (2)

where *ε_L_* is frequency-independent part of DP of the film, *ω* is the frequency of the incident light. *ω_p_* is the inherent plasma frequency of the rGO material associated with the carriers density *N_0_* at the interface, *m_eff_* is the carrier effective mass. Here, the above parameters are set as *ε_L_* = 8 (carbon material), *m_eff_* = 0.24 *m_e_* (*m_e_* the mass of electrons) and *D* = 100 nm. The thickness of the inhomogeneous rGO layer *a* can be estimated by the skin depth of the incident light, *a = λ_0_*/4*πk* = 800/(4*π* × 0.6) ≈ 0.1 μm, where *k* is the imaginary part of the dielectric constant of rGO. After introducing the aforementioned parameters into the dispersion relation, we can obtain the dispersion relation of TE-SP wave propagation along the rGO surface. The electric (*E*) field distribution can also be simulated by FDTD solution.

Generally, the inhomogeneous distribution of DP is critical to the generation of TE-SP, and the specific form is not strictly limited. To illustrate this point, we further designed an exponential distribution of DP for comparison. For example, when $\varepsilon\left( z \right)=a\cdot e^{b\cdot z}$, the *E* field distribution simulated by FDTD solution is shown in Figure S8, suggesting that the TE-SP can also be excited.


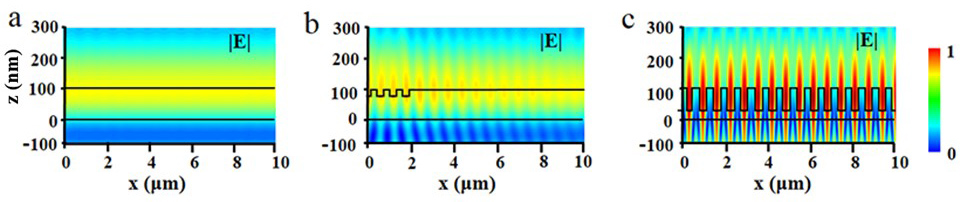


**Figure S8.** Simulated *E* field distribution of the rGO film without (**a**) and with (**b**, **c**) phase matching by introducing an *e*-exponential dependent DP variation. The depths of the initial and final structures are defined the same as Figure 3c.

**S9. Discussion of conditions for TE-SP wave excitation**

The dimensionless parameter *b* describes the inhomogeneity strength of the rGO film, which affects the gradual distribution of DP in *z* direction. Meanwhile, changing the value of the parameter *b* also makes different in the plasma frequency (*ω_p_*) and carriers density (*N_0_*) of the rGO material. Therefore, the excitation of TE-SP can be achieved in a large range of *b*, *ω_p_* and *N_0_*. Figure S9a and S9b show the *E* field distribution by introducing the maximum and minimum values of *b*, respectively. More possible parameters are shown in **Table S1**.


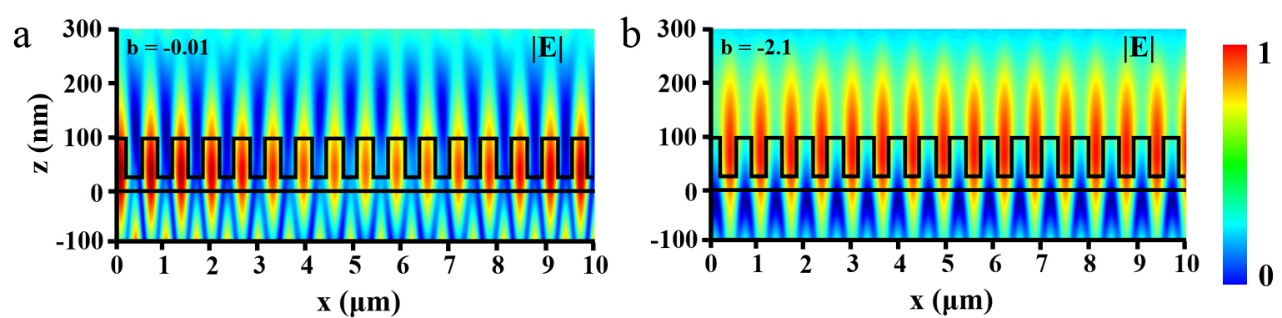


**Figure S9.** Simulated TE-SP excitation on the rGO film with both the maximum (**a**) and minimum (**b**) values of the parameter *b*.

**S10. Simulated *E* field distribution on rGO film with uniform DP in *z* direction**

We have already demonstrated that the inhomogeneous dielectric film is very necessary for the excitation of TE-SP. To further illustrate this point, we also simulated the *E* field distribution on the rGO film with the uniform DP distribution in *z* direction. It is clear that TE-SP cannot be excited even if the phase matching is introduced, as shown in Figure S10a and S10b.


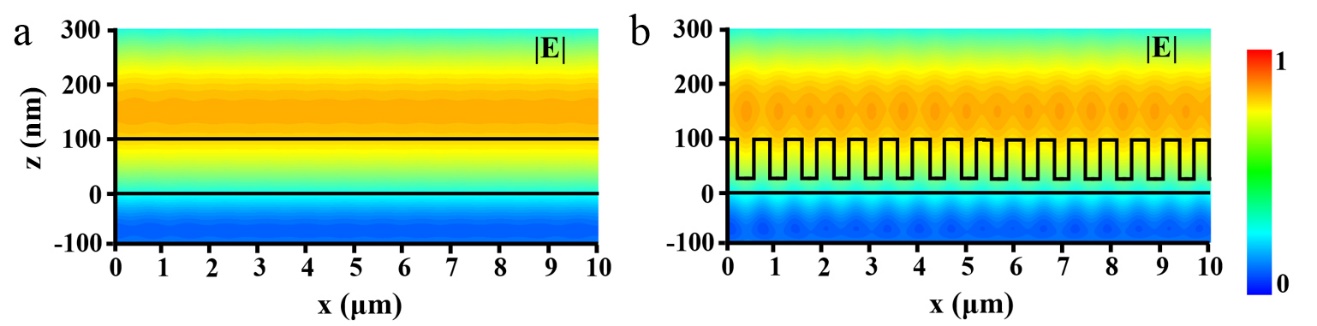


**Figure S10.** Simulated *E* field distribution on the rGO film without (**a**) and with (**b**) the phase matching when DP is uniform.

**S11. Simulated *E* field intensity distribution along *x* direction with different film thicknesses**

The strong-and-weak energy fringes with the certain spatial period were estimated on the rGO film. Here, we simulated intensity distributions of *E* field along the *x* direction for the films of different thicknesses (Figure S11). The spatial period of *E* field has little change regardless of how thick the film is in the period from 100 to 250 nm, which is consistent with the experimental results presented in Figure S3 (Λ = 680 ± 18 nm).


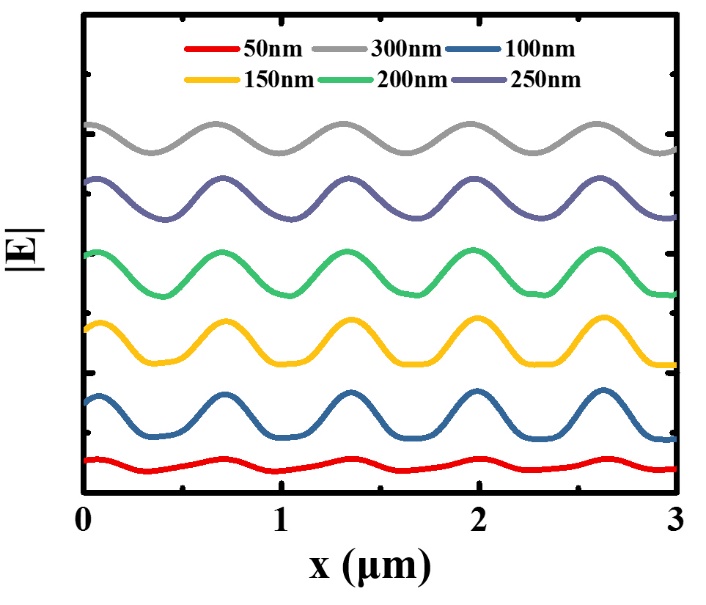


**Figure S11.** Simulated *E* field intensity distributions along the x direction on rGO films with several different thicknesses.

**S12. Simulated *E* field excitation by a TM-mode light**

Using the same parameters mentioned in the main text, we simulated the *E* field distribution on the rGO films under the laser irradiation with a TM mode, as shown in Figure S12. Although the spatially periodic distribution of the energy fringes can also been observed, indicating the excitation of TM-SP, their peak intensities are seen greatly reduced. We compared the *E* field intensity ($\left| E \right|$) distribution at the rGO-air interface, surface of 100 nm-thickness film marked by the red line in Figure S12a, excited by the incident TE and TM modes, respectively. It is obvious that the *E* field excited by the TE mode is much stronger. Therefore, it’s reasonable to think that, during the competition between TE-SP and TM-SP modes, the huge difference in the intensity of the mode excitation eventually makes the TE-SP predominant in the LIPSS formation.

**
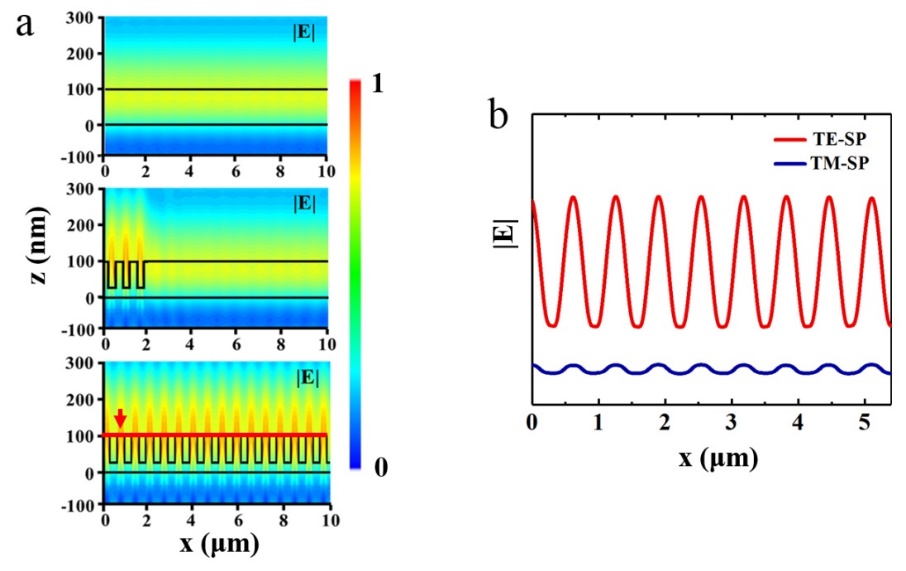
**

**Figure S12.** **a** Simulated *E* field distribution on the rGO film excited by the TM-mode incident light. **b** Simulated *E* field intensity distributions along the *x* direction at the rGO-air interface excited by TE and TM-mode incident light, respectively.**S13. Comparison of temperature responses for GO, thermal reduced GO (T-rGO) and rGO-LIPSS films**

We attribute the enhanced photoresponse of the rGO-LIPSS to its increasing absorption of the incident light by the surface structures. This can be confirmed by the change of temperature under illumination. To compare the effect of the reduction degree of the GO film on the light absorption, we also prepare a comparative sample using the thermal reduction method (T-rGO) without any induced surface structures. The optical microscopic images of three samples (GO, rGO-LIPSS and T-rGO) are presented in Figure S13a, respectively. Under the illumination of a LED light source, the situations of the temperature change for three samples were monitored by an infrared (IR) camera (Figure S13b). By comparison, we find that the rGO-LIPSS on the film can greatly enhance the photo absorption process, leading to a maximum temperature rise of ~10 °C compared with GO film. Meanwhile, for the case of T-rGO, the effect of the reduction degree of the GO film on the temperature rise is only ~1 °C, which in fact becomes negligible. This further indicates that the enhanced optical absorption of the sample is resulted from the surface structures rather than the reduction degree. Another fact is that the spatial regular distribution of the micro/nanostructures on the film play an important role in the optical absorption of materials. The more regular the structures, the higher the temperature rises, suggesting the stronger light absorption abilities.

**
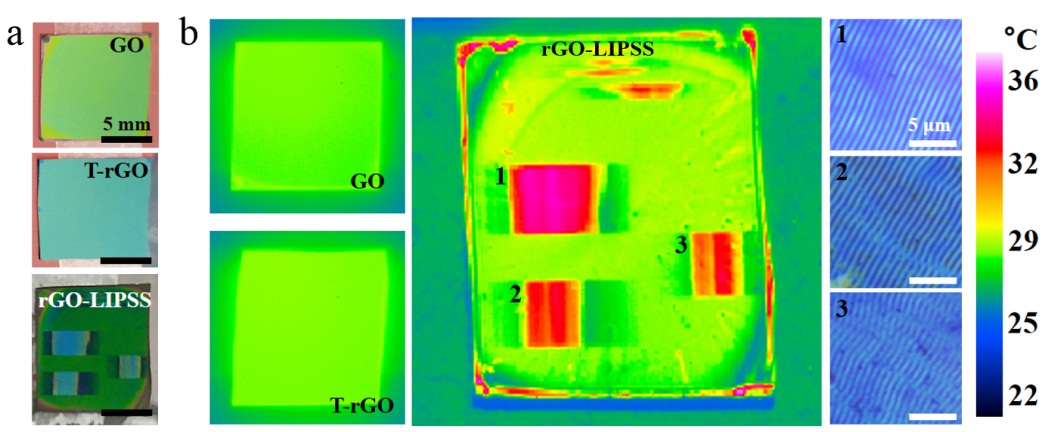
**

**Figure S13.** Experimental comparisons of temperature responses for GO, T-rGO and rGO-LIPSS samples**.** **a** Optical microscopic images. **b** IR images under the illumination of the LED light source, where 1, 2 and 3 stand for regular (1) and irregular (2, 3) rGO-LIPSS regions obtained under different laser parameters.

**Table S1. Discussion of conditions for TE-SP wave excitation**

When using values of the parameter *b* given in the following table, we can get the *E* field distributions through FDTD simulation, and the corresponding *N_0_* and *w_p_* are also calculated and shown in the table.

| ***b*** | ***w_p_*（×10^15^ s^-1^）** | ***N_0_* (×10^26^ m^-3^)** |  | ***b*** | ***w_p_*（×10^15^ s^-1^）** | ***N_0_* (×10^26^ m^-3^)** |
| --- | --- | --- | --- | --- | --- | --- |
| -0.01 | 0.25 | 0.05 |  | -0.8 | 1.55 | 1.82 |
| -0.05 | 0.5 | 0.19 |  | -1.1 | 1.67 | 2.11 |
| -0.1 | 0.75 | 0.43 |  | -2.1 | 1.85 | 2.6 |
| -0.4 | 1.25 | 1.18 |  |  |  |  |

**Table S2. Averaging ID/IG values with different samples**

We respectively choose the data from different Raman spectra of the GO film without any laser treatments, and at the ridge and valley positions on the rGO-LIPSS. Their specific values and the average I_D_/I_G_ ratios are listed in the following:

|  | GO | | | rGO-LIPSS-ridge | | | rGO-LIPSS-valley | | |
| --- | --- | --- | --- | --- | --- | --- | --- | --- | --- |
|  | ***I_D_*** | ***I_G_*** | ***I_D_/I_G_*** | ***I_D_*** | ***I_G_*** | ***I_D_/I_G_*** | ***I_D_*** | ***I_G_*** | ***I_D_/I_G_*** |
| Point 1 | 696 | 653 | 1.0658 | 165 | 174 | 0.9483 | 172 | 185 | 0.9297 |
| Point 2 | 678 | 636 | 1.066 | 165 | 172 | 0.9593 | 154 | 166 | 0.9277 |
| Point 3 | 702 | 657 | 1.0685 | 170 | 178 | 0.9551 | 158 | 171 | 0.924 |
| Point 4 | 724 | 680 | 1.0647 | 175 | 184 | 0.9511 | 167 | 180 | 0.9278 |
| Point 5 | 688 | 643 | 1.07 | 168 | 177 | 0.9492 | 158 | 170 | 0.9294 |
| Average |  |  | 1.067 |  |  | 0.952 |  |  | 0.928 |

**References**

1. Huang, M. *et al*. Origin of laser-induced near-subwavelength ripples: interference between surface plasmons and incident laser. *ACS Nano* **3**, 4062-4070 (2009).
2. Oktem, B. *et al*. Nonlinear laser lithography for indefinitely large-area nanostructuring with femtosecond pulses. *Nat. Photonics* **7**, 897-901 (2013).
3. Kim, K. Excitation of s-polarized surface electromagnetic waves in inhomogeneous dielectric media. *Opt. Express* **16**, 13354-13363 (2008).
